# Adipose-Derived Mesenchymal Stem Cells Migrate and Rescue RPE in the Setting of Oxidative Stress

**DOI:** 10.1155/2018/9682856

**Published:** 2018-12-13

**Authors:** Aya Barzelay, Shira Weisthal Algor, Anat Niztan, Sebastian Katz, Moshe Benhamou, Itay Nakdimon, Noam Azmon, Sandy Gozlan, Daphna Mezad-Koursh, Meira Neudorfer, Michaella Goldstein, Benjamin Meilik, Anat Loewenstein, Adiel Barak

**Affiliations:** ^1^Department of Ophthalmology, Tel Aviv Sourasky Medical Center, Tel Aviv, Israel; ^2^Sackler Faculty of Medicine, Tel Aviv University, Tel Aviv, Israel; ^3^Department of Plastic and Reconstructive Surgery, Tel Aviv Sourasky Medical Center, Tel Aviv, Israel

## Abstract

Oxidative stress leads to the degeneration of retinal pigment epithelial (RPE) and photoreceptor cells. We evaluated the potential of adipose-derived mesenchymal stem cells (ASCs) as a therapeutic tool by studying the migration capacity of ASCs *in vitro* and their protective effect against RPE cell death under oxidative stress *in vitro* and *in vivo*. ASCs exhibited enhanced migration when exposed to conditioned medium of oxidative stressed RPE cells obtained by hydrogen peroxide. Migration-related axis SDF-1/CXCR4 was studied, and upregulation of SDF-1 in stressed RPE and of CXCR4 in ASCs was detected. Moreover, ASCs' conditioned medium prevented H_2_O_2_-induced cell death of RPE cells. Early passage ASCs had high expression level of HGF, low VEGF levels, and unmodulated IL-1*β* levels, compared to late passage ASCs. Thus, early passage ASCs show the potential to migrate towards damaged RPE cells and protect them in a paracrine manner from cell death induced by oxidative stress. *In vivo*, mice received systemic injection of NaIO_3_, and 72 h later, ASCs were transplanted in the subretinal space. Seven days after ASC transplantation, the eyes were enucleated fixed and frozen for immunohistochemical analysis. Under such conditions, ASC-treated mice showed preservation of nuclear layers in the outer nuclear layer and stronger staining of RPE and photoreceptor layer, compared to PBS-treated mice. Taken together, our results indicate that ASCs are able to home in on damaged RPE cells and protect against damage to the RPE and PR layers caused by oxidative stress. These data imply the potential that ASCs have in regenerating RPE under oxidative stress, providing the basis for a therapeutic approach to retinal degeneration diseases related to oxidative stress that could help save the eyesight of millions of people worldwide.

## 1. Introduction

Retinal degeneration diseases related to oxidative stress and inflammation, such as age-related macular degeneration (AMD), are characterized with RPE injury and cell death [1–3]. Oxidative stress is associated with the release of reactive oxygen species as a result of high oxygen tension in the macula, phagocytosis of high concentrations of polyunsaturated fatty acids from photoreceptor outer segments by the retinal pigment epithelium (RPE), and finally, decreased antioxidant capacity with advanced age [4, 5]. Degeneration, apoptosis, and necrosis of the RPE are related to both inflammation and oxidative stress by several mechanisms [6–9]. Photoreceptors (PR), whose normal function and survival are strongly related to the activity of the RPE by various metabolic functions, such as phagocytosis of PR outer segments, are highly susceptible to oxidative damage due to their constant exposure to light and oxygen. Thus, RPE degeneration leads to secondary death of PR and their nuclei, concentrated in the outer nuclear layer (ONL).

Cell therapy for retinal degeneration diseases has recently gained attention. Among different sources of stem cells, adipose tissue-derived mesenchymal stem cells (ASCs) have emerged as a promising therapeutic modality, in light of their advantages: they can be easily isolated from subcutaneous fat by minimally invasive techniques [10], produced in larger quantities, as compared to bone marrow-derived mesenchymal stem cells [10], and additionally, characterized by high viability and reproducibility. Moreover, they serve as an autologous source for stem cells, thus precluding the need for immunosuppressive therapy, which may not be well tolerated by elderly patients [11]. Furthermore, ASCs, which bear migratory and homing abilities, also have immunomodulatory properties and secrete cytokines and growth factors [11, 12], thus facilitating replacement of dying cells, immunomodulatory capacity [12], and promotion of tissue remodeling and regeneration [11, 13]. Interestingly, mesenchymal stem cells have shown resiliency to oxidative stress, possibly due to low baseline levels of reactive oxygen species (ROS) and high levels of glutathione [14]. Therefore, this population of stem cells may serve as a therapeutic tool in the future treatment of retinal degeneration diseases that manifest with oxidative stress.

Taken together, ASCs may be a potential source of autologous stem cells with prosurvival and antioxidative activities. The aims of this study were to evaluate the migratory ability of ASCs under RPE-oxidative stress conditions, as well as the protective role of ASCs on RPE cell death and degeneration of surrounding tissues.

## 2. Materials and Methods

The purpose of the study and the procedures used were presented to all of the subjects, and a signed informed consent was obtained from each. This study was approved by the ethics committee for clinical trials of Tel Aviv Sourasky Medical Center, and the procedures used conformed to the tenets of the Declaration of Helsinki.

### 2.1. Isolation, Characterization, and Culture of ASCs

#### 2.1.1. Isolation and Culture of Human Adipose Tissue-Derived Stem Cells (ASCs)

Human adipose tissue was harvested from 5 healthy patients with a mean age of 38 ± 4.3 and body mass index of 28.2 ± 3.9 who had abdominoplasty for aesthetic reasons at Tel Aviv Sourasky Medical Center. No metabolic diseases, HIV, hepatitis, or other systemic complications were reported from these patients.

The isolation and culture of ASCs were performed as previously described [15]. Briefly, 60 to 120 ml of the raw lipoaspirates was washed with phosphate-buffered saline (PBS) and enzymatically digested with 0.75% collagenase type I (Cat. no. C1639, Sigma) at 37°C for 1 hour. The digested lipoaspirates were centrifuged at 400 g for 15 minutes, and the pellet was resuspended and passed through a 100 *μ*m mesh filter (Cat. no. 542000, EASYstrainer, Greiner Bio-One) to remove debris. Subsequently, 1 × 10^6^ cells were plated in 100 mm culture dishes in ADSC medium and incubated at 37°C in a humidified 8% CO_2_ atmosphere [16]. The medium was changed twice weekly, and cells were passaged with 0.25% trypsin/0.1% EDTA (Biological Industries, Israel) upon reaching 90% confluency. Experiments were performed at passages 3–4.

#### 2.1.2. Characterization of ASCs for Mesenchymal Stem Cell (MSC) Markers by Immunostaining and FACS Analysis

Characterization of cultured ASCs was performed at passage three as follows: after reaching 100% confluence, cells were trypsinized and collected in FACS tubes in aliquots (1 × 10^5^ cells/tube). Cells were then stained with fluorescein isothiocyanate (FITC) and phycoerythrin- (PE-) conjugated monoclonal antibodies against human CD34 (Dako), CD45 (Dako), CD90 (Dako), CD105 (eBioscience), and CD73 (BD Pharmingen). Cells were subsequently analyzed by FACS Canto II flow cytometer (BD Biosciences). Isotype-matched FITC and PE-conjugated antibodies were used as controls.

#### 2.1.3. Multipotency of ASCs by Differentiation to Osteocytes and Adipocytes

ASCs at passage 3 were studied for their ability to differentiate to osteocytes and adipocytes. Cells were seeded in a 24-well plate at a density of 1 × 10^4^ cells per well. At confluency of 100%, differentiation media were added to the cells and changed twice a week (adipose: 10% FBS, 1 *μ*M dexamethasone, 0.5 mM 3-isobutyl-1-methylxanthine, 10 *μ*g/ml insulin, and 100 *μ*M indomethacin in high glucose (HG) DMEM; bone: StemPro® osteocyte differentiation basal medium (Gibco)). Protocol lasted either two or three weeks to induce bone and adipose differentiation, respectively.

Differentiation to adipocytes was assessed using an Oil Red O stain as an indicator of intracellular lipid accumulation. The cells were fixed for 20 min at room temperature in 4% paraformaldehyde. Cells were incubated in 0.5% (wt/vol) Oil Red O reagent in 100% Isopropanol (Sigma) for 10 min at room temperature. Excess stain was removed by washing with distilled water.

Bone differentiation was assessed using Alizarin red (Sigma). Cells were fixated with 4% paraformaldehyde for 20 min and then stained with Alizarin red 2% solution adjusted to pH 4.2 for 15 min at room temperature. Excess stain was removed by washing with several changes of distilled water.

Images of stained cells with both Oil Red O and Alizarin red were taken by light microscopy. Results are presented as the percent of stained cells from the total number of cells counted in a high-power field.

### 2.2. Primary RPE Culture

5.5 × 10^5^ cells of human pRPE cells (Lonza) were plated in 100 mm culture dishes (Falcon) in RtEGM BulletKit Medium (Lonza) and incubated at 37°C in a humidified 5% CO_2_ atmosphere. The medium was replaced twice weekly, and cells were passaged with 0.25% trypsin/0.1% EDTA (Biological Industries, Israel) upon reaching 90% confluence. Experiments were performed at passages 3–4.

### 2.3. Scratch Assay

#### 2.3.1. Oxidative Stress Induction

RPE cells were seeded at 1 × 10^4^ cells/cm^2^ in RtEGM medium containing 2% FBS (Lonza). After adhesion of the cells to the dish, the medium was changed to FBS-free RtEGM and renewed every two days until treatments. To induce oxidative stress, subconfluent RPE cells were treated for 16 h with 0.5 mM H_2_O_2_ (Cat. no. 216763, Sigma) in ADSC serum-free medium; the medium was collected and centrifuged at 1500 rpm for 5 min, and the supernatant was collected (stressed RPE-CM).

#### 2.3.2. Scratch Assay

ASCs were seeded in 6-well plates (Falcon) until confluence. Cells were cultured in ADSC serum-free conditions to prevent proliferation [17], and the monolayers were then scored with a sterile pipette tip to leave a scratch. The culture medium was immediately removed along with any detached cells and replaced with either fresh ADSC serum-free medium (non-CM), stressed RPE-CM, or conditioned medium of RPE cells not treated with H_2_O_2_ (RPE-CM). All scratch assays were performed in quadruplicates, and images were taken at the beginning of the treatments (time zero) and after 24 h (H_2_O_2_ treatments). The number of cells migrating to the scratched area was counted under high-power magnification and in a blinded fashion. ASCs as well as RPE cells were then harvested for mRNA analysis by qRT-PCR.

### 2.4. Quantitative RT-PCR

Total RNA was extracted from ASCs or RPE cell cultures using High Pure RNA Isolation Kit (Roche) according to the manufacturer's instructions. Total RNA concentration was determined by NanoDrop™ 1000 Spectrophotometer (Thermo Scientific) and was reverse transcribed using Verso cDNA synthesis kit (Thermo Scientific). The mRNA expression levels of the growth factors, hepatocyte growth factor (HGF), vascular endothelial growth factor (VEGF), interleukin-1*β* (IL-1*β*), stromal-derived factor-1 (SDF-1), the chemokine receptor CXCR4, and normalizing housekeeping genes GUSB and RLP27 (see Table S1 for sequence information) were measured by real-time reverse transcription polymerase chain reaction (RT-PCR) (StepOnePlus, Applied Biosystems) using SYBR® Green qPCR Mastermix (Qiagen). The cycling RT-PCR conditions were as follows: 10 min at 95°C, 40 cycles for 10 s at 95°C, 15 s at 60°C, followed by gradient stage from 60 to 95°C to obtain a melting curve. The results were calculated by the ΔΔCT method of relative quantitation.

### 2.5. Rescue Studies

#### 2.5.1. Preparation of ASC Conditioned Medium

ASCs (1 × 10^6^ cells/cm^2^) at passage 3 or passage 5 were seeded on a 100 mm dish (Falcon) and cultured in ADSC BulletKit™ Medium (Lonza). At 100% confluence, ASCs were washed with phosphate-buffered saline (PBS) and cultured with ADSC serum-free medium (Lonza) for 48 h. Medium was collected, filtered using a 0.22 mm syringe filter, and either immediately transferred to RPE cells or maintained in −80°C for further protein analysis using ELISA assay. In turn, ASCs were harvested for mRNA level detection using RT-PCR. ASCs at passage 5 showed aspects of senescence evident by low proliferation rate and morphology changes (data not shown). The condition medium of ASCs at passage 5 (P5-CM) was used in this study as negative control to the condition medium collected from ASCs at passage 3 (P3-CM).

#### 2.5.2. Rescue Study

RPE was preincubated with ASC-CM followed by treatment with H_2_O_2_. RPE cells were seeded as described above in a 6-well plate (Falcon); after reaching approximately 90% confluence, RPE cells were preincubated for 48 h with either P3-CM, P5-CM, or with nonconditioned ADSC serum-free medium (non-CM). RPE cells were then washed with PBS followed by exposure to 1 mM H_2_O_2_ or without H_2_O_2_ as a control. After 7 h, RPE cell death was monitored by propidium iodide (PI) using FACS analysis and by ethidium bromide and acridine orange fluorescent staining.

#### 2.5.3. Propidium Iodide Staining and Flow Cytometry Analysis

Following rescue studies as described above, RPE cells (3 × 10^5^) at passage 3 were harvested with 0.25% trypsin/EDTA (Biological Industries). Cells were collected by centrifugation at 500g for 5 min, washed twice with PBS, and resuspended in 400 *μ*l of PBS to which 1 *μ*l of propidium iodide (Sigma) 1 mg/ml was added immediately before flow cytometry measurements. At least 10,000 events were collected and labeled; fluorescence cells were detected by BD FACS Canto^™^ II cytometer (BD Pharmingen, USA). Analysis of cell death distribution was conducted by FCS Express 4 software (De Novo Software, Canada).

#### 2.5.4. Ethidium Bromide and Acridine Orange Fluorescent Staining

Following rescue studies as described above, RPE cells were collected by trypsinization; the apoptosis and necrosis rates of RPE were assessed using ethidium bromide and acridine orange fluorescent staining as follows: fluorescent staining solution (0.5 *μ*l) containing an equal volume of 100 *μ*g/ml acridine orange and 100 *μ*g/ml ethidium bromide (Sigma) was added to each cell suspension sample and then covered with a coverslip. 150,000 cells were counted and the morphology of cell death was examined and analyzed immediately at room temperature.

### 2.6. Enzyme-Linked Immunosorbent Assay (ELISA)

Conditioned media were prepared as described above; briefly, ASCs' medium was changed to serum-free medium; following 48 h of incubation, medium was collected and centrifuged at 2500 rpm for 5 min, filtered, and stored at −80°C until it was assayed. Levels of HGF were measured by ELISA according to manufacturer protocols (HGF, R&D Systems), and results were compared to the control group comprising senescent ASCs evident by high passage, phenotype, and low proliferation.

### 2.7. Animal Procedure

Wild-type C57BL mice received intraperitoneal (IP) injection of 50 mg/kg of sodium iodate (NaIO_3_) (Sigma-Aldrich) (*n* = 8 mice in each group, ASC treatment versus PBS). The sodium iodate model manifests oxidative stress, acute injury to RPE, and consequently progressive ongoing retinal damage [18, 19]. Seventy-two hours after injection, mice were anesthetized and a small self-sealing sclerotomy was performed with the tip of a 30-gauge needle. A 33-gauge needle attached to a Hamilton syringe (Hamilton Company, Reno, Nevada, USA) was inserted through sclerotomy into the subretinal space, and an injection of 1.5 *μ*l of PBS containing 4 × 10^4^ cells ensued. The injection was unilateral in all animals. To note, mice received cyclosporine in drinking water after the transplantation, at a concentration of 210 mg/l [20]. Animal handling and experiments were performed following institutional care guidelines with the approval of the Tel Aviv Sourasky Medical Center Animal Ethics Committee.

### 2.8. Tissue Preparation

One week following subretinal injection, mice were euthanized; the eyes were enucleated and fixed in 3.7% formaldehyde (Merck, Darmstadt, Germany) in PBS overnight. The eyes were then infiltrated for cryoprotection with 5% sucrose (Sigma-Aldrich) in PBS at room temperature for 1 h, followed by 30% sucrose in PBS overnight at 4°C. Fixed tissue was embedded in OCT Tissue Freezing Medium (Scigen Scientific, Gardena, CA, USA) and frozen on dry ice. Cross-sections (10 *μ*m) were placed on X-tra adhesive slides (Leica Biosystems, Peterborough, UK) and stored at −20°C.

### 2.9. Immunocytochemical Analysis

Sections were washed in PBS for 20 minutes and blocked with 1% bovine serum albumin (BSA) (Sigma-Aldrich), 10% normal goat serum (NGS) (Jackson ImmunoResearch, Baltimore, MD), and 0.25% Triton X-100 (Sigma-Aldrich) in PBS for 1 h at room temperature. The sections were incubated overnight with the primary antibodies in blocking solution at 4°C. The slides were washed three times with PBS, incubated with the secondary antibodies for 1 h at room temperature, and washed again three times with PBS. The sections were then incubated with the nuclear dye DAPI (Molecular Probes, Thermo Fisher Scientific) for 10 minutes, washed once in PBS, and mounted with ProLong Gold antifade reagent (Invitrogen, Thermo Fisher Scientific). Observations and photography were carried out with a Zeiss LSM 510 confocal microscope in a blinded manner. Analysis was carried out by ImageJ software. ONL thickness was assessed by counting the number of nuclei rows at different points along the retinal length [21]. RPE65 intensity was quantified by marking the RPE layer and measuring the fluorescence intensity compared to the image's background using ImageJ software. The size of the PR layer was quantified by measuring the width of the layer at different points along the layer using ImageJ software.

#### 2.9.1. Primary Antibodies

Mouse anti-rhodopsin monoclonal antibody, 1 : 200 (EMD Millipore, Merck, Darmstadt, Germany), and anti-RPE65 antibody, mouse monoclonal, ab78036 1 : 500 (Abcam), were used.

#### 2.9.2. Secondary Antibodies

Alexa Fluor 488 Goat anti-mouse IgG and Alexa Fluor 546 Goat anti-mouse IgG were used. All the secondary antibodies (Invitrogen, Thermo Fisher Scientific) were diluted 1 : 400 in PBS.

### 2.10. Statistical Analysis

For the *in vitro* experiments, each experiment was performed a minimum of 3 samples from 3 different patients. Each experiment was performed a minimum of 3 times. For the *in vivo* experiments, each experimental group included 8 mice. Statistical analysis was performed using paired *t*-test. *p* value ≤ 0.05 was considered statistically significant.

## 3. Results

### 3.1. Phenotypic Characterization and Multipotency of ASCs

**Figure 1:**
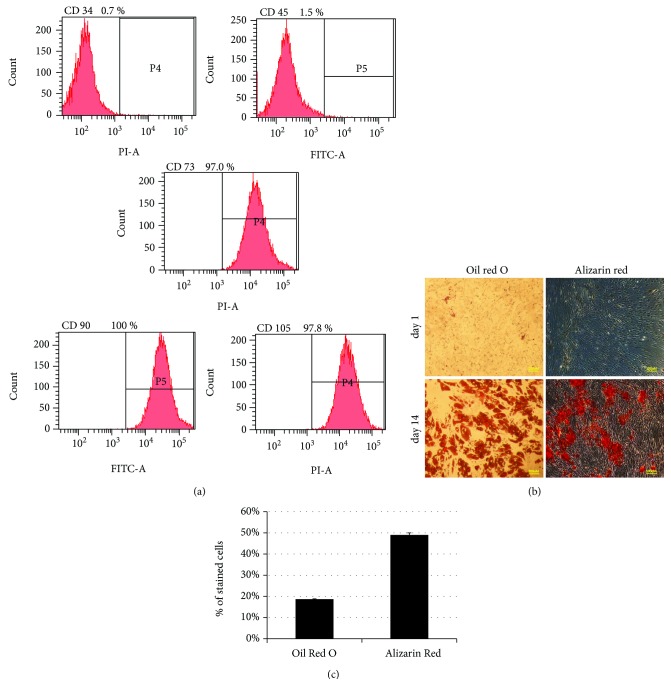
Characterization of ASCs by surface phenotype and differentiation potential at passage 3. Cultured ASCs at passage 3 were detached with trypsin, equally dispensed into FACS tubes (1 × 10^5^ cells per tube), and incubated with monoclonal antibodies against human CD34, CD45, CD90, CD73, and CD105. Cells were then analyzed by flow cytometry for the expression of cell surface markers. CD: cluster of differentiation. Each experiment was performed a minimum of 3 samples from 3 different patients. Each experiment was performed a minimum of 3 times.

ASCs were isolated from lipoaspirate of donors' subcutaneous fat. Phenotypic characterization was studied at passage 3 using immunostaining and FACS analysis. ASCs expressed classic MSC markers (CD90: 100 ± 1.98, CD73: 97 ± 5.2, and CD105: 97.8 ± 1.7% of population) and were negative for hematopoietic markers (CD45:1.5 ± 0.9, CD34:0.7 ± 0.6) (Figure 1). ASCs exhibited multipotency evident by their ability to differentiate into osteocytes and adipocytes, as evidenced by the percentage of cells stained with Alizarin red and Oil Red O, respectively (Figures 1(b) and 1(c)).

### 3.2. Early Passage ASCs Express High Levels of the Neurotropic Protein HGF but Not the Angiogenesis-Related VEGF Factor and Proinflammatory Cytokine IL-1*β*

**Figure 2:**
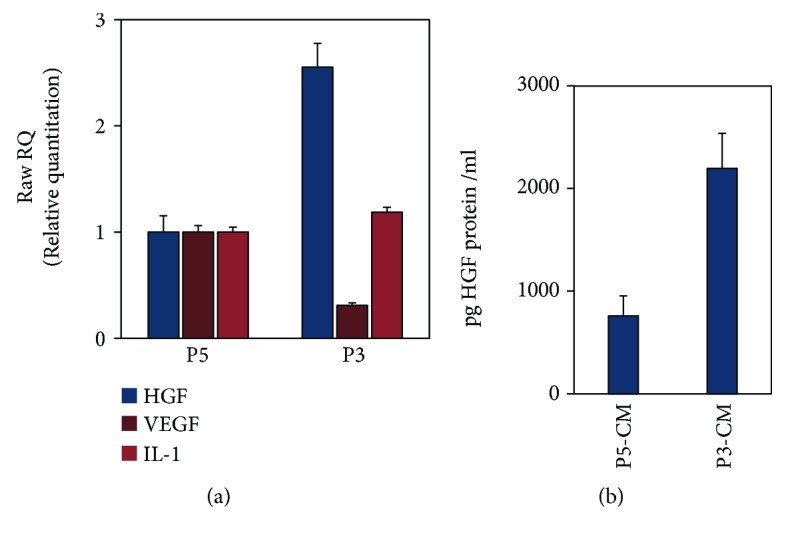
Early passage ASCs overexpress the neurotropic protein HGF but not VEGF nor the proinflammatory cytokine IL-1*β*. ASCs at passage 3 that were cultured in serum-free conditions for 48 hours were compared to the control group of ASCs at passage 5. Both cells and medium were collected and analyzed at mRNA level and at protein level by qRT-PCR and by ELISA, respectively. (a) qRT-PCR analysis of HGF, VEGF, and IL-1*β*. (b) ELISA for HGF protein levels. Each experiment was performed a minimum of 3 samples from 3 different patients. Each experiment was performed a minimum of 3 times.

Examination using quantitative RT-PCR of the expression levels of several cytokines and growth factors secreted by ASCs revealed that ASCs at passage 3 (P3), compared to passage 5 (P5), showed consistently high levels of HGF, lower levels of VEGF, and unchanged expression levels of IL-1*β* (HGF: 2.55 ± 0.26-fold, VEGF: 0.31 ± 0.14-fold, and IL-*β*: 1.18 ± 0.1-fold) (Figure 2(a)). The expression of HGF was further validated at the protein level by ELISA and was demonstrated to increase by 2.9-fold at P3 compared to P5 (Figure 2(b)).

### 3.3. Enhanced Migration of ASCs following Exposure to Stressed RPE-CM Corresponds to SDF-1 and CXCR4 Upregulation in RPE Cells and ASCs, Respectively

**Figure 3:**
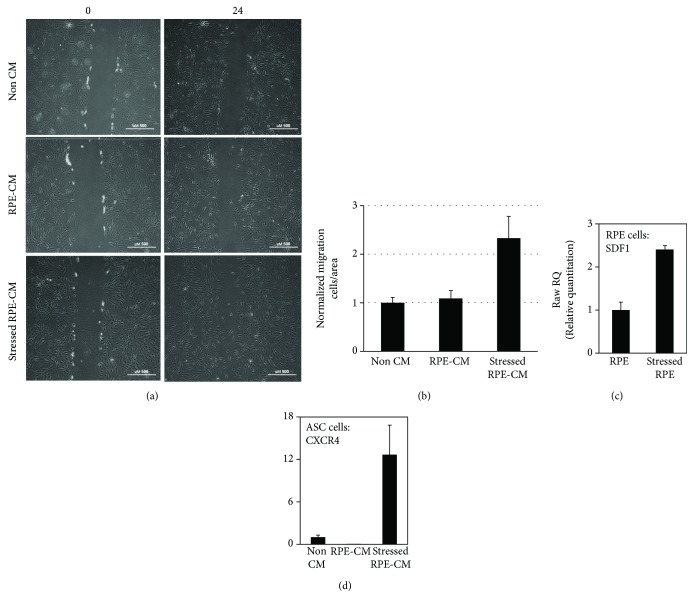
Enhanced migration of ASCs following exposure to stressed RPE-CM corresponds to SDF-1 and CXCR4 upregulation in RPE and ASCs, respectively. The migratory ability of ASCs was studied by scratch assay after exposure to stressed RPE-CM (RPE treated with H_2_O_2_) or to controls comprising ASCs exposed to RPE-CM (RPE cultured without H_2_O_2_) and non-CM (nonconditioned ADSC medium). (a) ASCs were monitored at 0 and 24 hours postscratch (×10 magnification). (b) Quantification of ASCs' migration by counting invasive cells in scratch boundaries. All scratch assays were performed in quadruplicates, and images were taken at the beginning of the treatments (time zero) and after 24 h (H_2_O_2_ treatments). ASCs and RPE cells were harvested and mRNA levels were analyzed using RT-PCR. (c) SDF-1 mRNA in RPE cells incubated with or without H_2_O_2_. (d) CXCR4 mRNA in ASCs incubated with stressed RPE-CM, RPE-CM, or non-CM. CXCR4: chemokine receptor type 4; SDF-1: stromal cell-derived factor 1; RPE: retinal pigment epithelium; ASCs: adipose-derived stem cells; CM: conditioned medium.

As described above, ASCs were exposed to non-CM, RPE-CM, and stressed RPE-CM and their migration was assessed. ASC migration capacity was significantly enhanced upon exposure to stressed RPE-CM as opposed to exposure to RPE-CM or non-CM (2.33- and 2.14-fold increase, respectively, *p* value < 0.05) (Figures 3(a) and 3(b)).

Using qRT-PCR, we analyzed the expression levels of SDF-1 and CXCR4. In stressed RPE cells, SDF-1 was significantly upregulated when compared to the expression of SDF-1 in nonstressed RPE cells (2.4 ± 0.09-fold) (Figure 3(c)). Accordingly, exposure of ASCs to stressed RPE-CM resulted in the upregulation of the SDF-1 receptor, CXCR4, compared to non-CM (12.6 ± 4.5-fold) (Figure 3(d)).

### 3.4. ASCs Inhibit RPE Cell Death under Oxidative Stress

**Figure 4:**
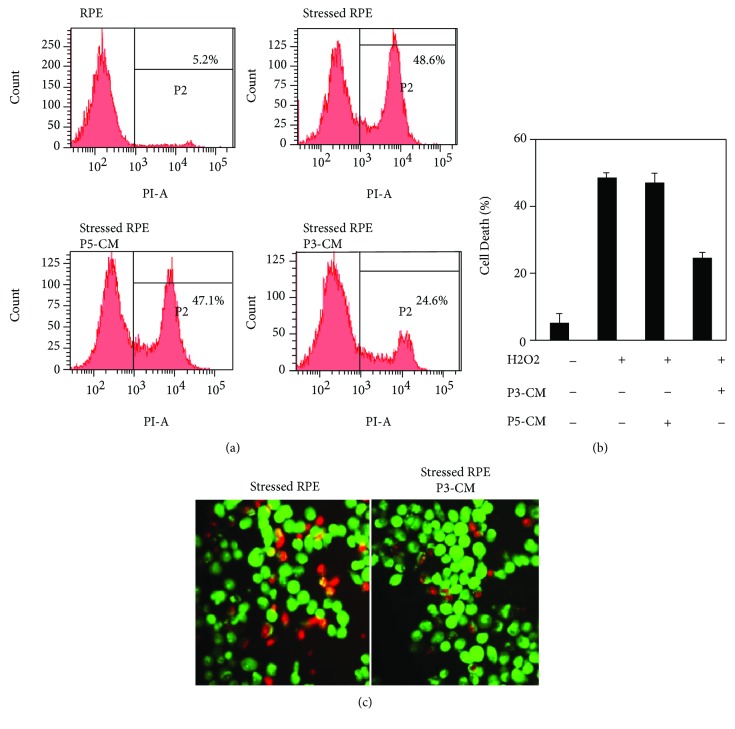
ASCs rescue RPE cell death under oxidative stress. RPE cells were incubated with P3-CM or with controls comprising P5-CM or non-CM for 48 hours, followed by exposure to H_2_O_2_ (1 mM, 7 h). Cells were harvested and cell death was analyzed. (a, b) Necrotic cell death was determined using PI staining followed by flow cytometry analysis. (c) Cell death visualized by acridine orange and ethidium bromide staining. Live cells appear green stained by acridine orange only (×20 magnification). CM: conditioned medium. Each experiment was performed a minimum of 3 samples from 3 different patients. Each experiment was performed a minimum of 3 times.

Next, we assessed the protective role of ASC-CM on RPE cells exposed to H_2_O_2_. RPE cells were preincubated for 48 h with either ASCs' conditioned medium at passage 3 (P3-CM), ASCs' conditioned medium at passage 5 (P5-CM), or nonconditioned ADSC serum-free medium (non-CM), followed by H_2_O_2_ (1 mM, 7 h) treatment. RPE cells exposed to P3-CM prior to H_2_O_2_ treatment exhibited a significant decrease in cell death compared to non-CM, as evidenced by FACS analysis for propidium iodide (50.6% ± 1.6 cell death reduction), while P5-CM had no effect on cell viability (Figures 4(a) and 4(b)). Furthermore, stressed RPE cells rescued by P3-CM were also detected by ethidium bromide and acridine orange staining assay (51.5% cell death reduction from total cell death counted in RPE cells exposed to H_2_O_2_ only) (Figure 4(c)).

### 3.5. Sodium Iodate Induces RPE Damage

To examine the potential of ASCs in repairing damage caused by oxidative stress, adult C57BL mice were injected with sodium iodate (NaIO_3_), which serves as a model for acute RPE injury and retinal degeneration by inducing oxidative stress [19] and progressive ongoing damage [18] [22]. NaIO_3_ was injected intraperitoneally and induced damage to the RPE and PR layers in all mice examined, compared to mice that did not receive NaIO_3_ (data not shown).

### 3.6. Effect of ASC Treatment on the RPE Layer

**Figure 5:**
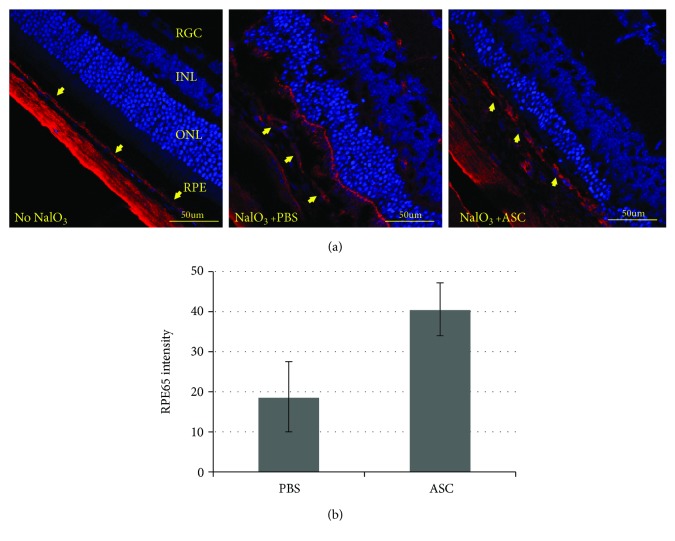
Retinal pigment epithelium layer. (a) Retinal slices were stained with RPE65 antibody (red) and the nuclear dye DAPI (blue). (b) RPE65 intensity was quantified by marking the RPE layer and measuring the fluorescence intensity compared to the image's background using ImageJ software. RPE layer staining was stronger in the eyes that received treatment with ASCs (*p* value = 0.0073). Yellow arrows indicate the RPE layer. Bar = 50 *μ*m. ONL: outer nuclear layer; INL: inner nuclear layer; RGC: retinal ganglion layer. *n* = 8 for each studied group.

The eyes treated with ASCs showed significantly higher levels of RPE65 staining compared to PBS-treated eyes, indicating a protective effect of ASCs from NaIO_3_-induced damage (Figure 5).

### 3.7. Effect of ASC Treatment on the PR Layer

**Figure 6:**
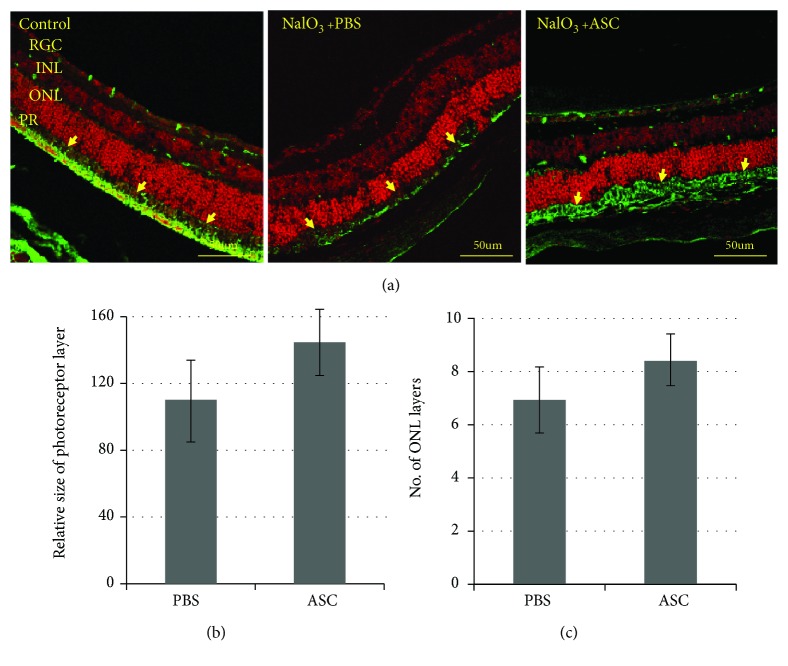
Photoreceptor layer size, rhodopsin labeling intensity, and ONL thickness. NaIO_3_ injection damages the RPE layer that nourishes the photoreceptor layer. Rhodopsin is a light-sensitive protein found in rod cells. (a) Retinal slices were stained with propidium iodide (nuclear dye) and rhodopsin antibody (green). The size of the photoreceptor layer (b) was measured by ImageJ software. The photoreceptor layer in the retinas treated with ASCs was larger compared to that in the PBS-treated retinas (*p* value = 0.0505). (c) ONL thickness was assessed by counting the number of nuclei rows at different points along the retinal length. ASC-treated eyes had significantly more photoreceptor cells than PBS-treated mice (*p* value = 0.0013). Bar = 50 *μ*M; yellow arrows indicate the PR layer. PR: photoreceptor layer; ONL: outer nuclear layer; INL: inner nuclear layer; RGC: retinal ganglion layer. *n* = 8 for each studied group.

NaIO_3_ injection damages the RPE layer that nourishes the PR layer. The size of the PR layer in the eyes treated with ASCs was larger compared to that in the PBS-treated eyes (Figures 6(a) and 6(b)), indicating that ASC treatment prevents damage not only to the RPE layer but also to the PR layer.

### 3.8. Effect of ASC Treatment on ONL Thickness

ONL thickness was positively affected in ASC-treated mice, evident in the preservation of nuclear layers of the ONL in ASC-treated mice when compared to the PBS-treated group (6.94 ± 1.2 ONL layers in PBS-treated mice, 8.4 ± 1.0 ONL layers in ASC-treated mice, *p* value = 0.0013) (Figure 6(c)).

## 4. Discussion

Oxidative stress has been shown to be one of the cardinal pathogenesis of RPE damage in several retinal degenerative diseases [3, 23], leading to the degeneration of the RPE and subsequently to PR loss [1, 2, 8]. ASC-based regenerative therapy for RPE shows great promise in providing a protective effect from oxidative stress-induced RPE cell death.

In this study, we demonstrated that oxidative stressed RPE-CM promoted ASC migration capacity, possibly by activating the migration associated-SDF-1/CXCR4 axis.

We also demonstrated that ASCs were able to prevent primary human RPE cell death caused by oxidative stress induced with H_2_O_2_. Interestingly, ASC-CM inhibition of RPE cell death was coupled with upregulation of the prosurvival HGF growth factor in RPE cells. Finally, transplantation of ASCs to the subretinal space in NAIO_3_ mice resulted in the preservation of RPE and photoreceptor layers with mild preservation of the ONL at one week.

SDF-1 is a chemo-attractant known to be involved in the migration and homing of stem cells [24]. Under chronic hypoxia and oxidative stress, MSCs were shown to express the SDF-1 receptor CXCR4 *in vivo* and *in vitro*, suggesting that SDF-1 serves as a mediator in the migration and homing of MSCs [14, 24]. This study reports that consequent to incubation of ASCs with stressed RPE-CM, CXCR4 is upregulated in ASCs as a possible response to SDF-1 overexpression in oxidative stressed-RPE cells. However, this study did not examine the direct correlation between an upregulation of SDF-1/CXCR4 expression and ASC migration or the direct effect between the migration *in vitro* and the protective effect of ASCs seen here *in vivo*. This enhanced migratory capability of ASCs upon exposure to oxidative stressed RPE-CM could provide the basis for an important future study, examining whether ASCs have a potential of homing to the site of injury once transplanted *in vivo*.

The retina is highly susceptible to oxidative stress through the increase in reactive oxygen species (ROS) followed by RPE cell death, leading to photoreceptor degradation, which construct the pathophysiology of retinal degeneration [23]. Although it is recognized that RPE apoptosis is one of the major events in the pathogenesis of retinal diseases, several reports have demonstrated RPE cell death not only via apoptosis [7] but also necrosis and necroptosis [9, 25]. In the current study, the exposure of RPE to H_2_O_2_ induced RPE cell death, while preincubation of RPE cells with ASC-conditioned medium reduced remarkably RPE cell death caused specifically by oxidative stress. The ability of ASCs to prevent cell death was also demonstrated by Singh et al. [26]; however, cell death was induced by the proliferation inhibitor, chemotherapeutic drug, Mitomycin C, i.e., via mechanisms different from oxidative stress and using the commercially immortalized cell line ARPE19 which is known to have lost some of the key features of primary RPE [27]. Furthermore, we show that P3 ASCs overexpress the neurotropic growth factor HGF compared to P5 ASCs. HGF is well linked to the MSC regenerative activity [28, 29] and was shown to protect RPE from oxidative stress-induced cell death by various mechanisms [30, 31]. To note, it was reported that HGF correlated with CNV progression in animal laser model of choroidal neovascularization (CNV) [32]; however, clinical data failed to exhibit HGF levels in vitreous of AMD patients [33–35]. Here, we demonstrated that an increase in HGF levels in ASC-CM correlated with ASCs' rescue of RPE from cell death, as the protective effect was abolished in the control group of ASCs at passage 5 which exhibited low HGF expression accompanied with no rescue effect on RPE. Similarly, the results of our animal experiments, in which we used P3 ASCs, provide further support to the protective, and potentially regenerative, capabilities of ASCs, possibly mediated via secretion of HGF.

Concomitant with HGF overexpression by P3 ASCs, we further demonstrate that P3 ASCs express significantly lower levels of VEGF compared to P5 ASCs. To be noted is that although a CD34+/CD90+ subpopulation of ASCs was shown to express high levels of VEGF [13, 36], ASCs used in this research were spontaneously CD34 negative, in line with low expression level of VEGF. Elevated VEGF secretion is known to contribute to additional deterioration in AMD [37]. Finally, the proinflammatory cytokine IL-1*β* remained unmodulated between P3 and P5. IL-1*β* was shown to be a neurotoxic and proinflammatory mediator in the retina [38]. High HGF secretion, accompanied with low or unmodulated VEGF and IL-1*β* levels, is a highly valued property in *in vivo* transplantation of ASCs.

Indeed, our mouse model for acute injury to RPE combined with oxidative stress [19, 39] provided further support for ASCs' ability to protect the RPE and PR layers from oxidative stress damage. The stronger staining of RPE65 in the RPE layer, as well as the larger size of the PR layer, compared to PBS treatment indicates that the damage induced by NaIO_3_ was significantly reduced in the eyes treated with ASCs. Moreover, the ONL layer thickness was preserved in ASC-treated mice. However, to note, these data relate to a short treatment of 1 week, and the effect seen in RPE, PR, and ONL layers did not translate to a full recovery in retinal morphology to a healthy state. Thus, future study will include observation of the therapeutic effect of ASCs after a longer time *in vivo* as well as the addition of functional studies such as ERG recordings and fundus photographs to better characterize the protective effect shown here.

Thus, taken together, our results demonstrate the potential of ASCs in treating stressed RPE by acting in two distinct manners, one by protecting the RPE cells from oxidative stress damage and the other by preventing further damage, possibly via their regenerative capabilities, as indicated in our animal experimentation. Combined with the increase in ASCs' migration *in vitro* in response to stressed RPE medium, ASCs hold great promise as a potential therapeutic approach to treat retinal pathologies in which RPE cells suffer an oxidative stress and cell death, eventually allowing millions of people worldwide to maintain their eyesight.

## Data Availability

The entire data used to support the findings of this study are included within the article.

## Supplementary Materials

Supplementary Materials Table S1: primers used in quantitative RT-PCR experiments.
