# Supplementary material for: Adipose-Derived Mesenchymal Stem Cells Migrate and Rescue RPE in the Setting of Oxidative Stress
Source: Stem Cells Int. 2018 Dec 13;2018:9682856. doi: 10.1155/2018/9682856 (PMC6311721; doi:10.1155/2018/9682856)
Supplement: Supplementary Materials — Table S1: primers used in quantitative RT-PCR experiments. [file 9682856.f1.docx]

**Supplementry material**

**Table S1. List of human primers used in this study**

| Sequence (5' to 3') R | Sequence (5' to 3') F | Human Gene |
| --- | --- | --- |
| CTACCTCCACCATGCCAAGT | GCAGTAGCTGCGCTGATAGA | VEGF |
| CCCCTTCAATAGCATGTCAA | GCTGTGTTCGTGTGGTATCAT | HGF |
| ATACGGAGCCCCCTTGTCT | CCTGCGTCCCACCTAGAATC | GUSB |
| AACAGGCTGCTCTGGGATTC | AGTCATCCTCATTGCCACTGT | IL-1β |
| ATCGCCAAGAGATCAAAGATAA | TCTGAAGACATCCTTATTGACG | RLP27 |
| TTCGGGTCAATGCACACTTG | TCTTCGAAAGCCATGTTGCC | SDF1 |
| TATCTGTGACCGCTTCTACC | GCAGGACAGGATGACAATAC | CXCR4 |
